# Mechanisms of LPS-Induced Acute Kidney Injury in Neonatal and Adult Rats

**DOI:** 10.3390/antiox7080105

**Published:** 2018-08-08

**Authors:** Egor Y. Plotnikov, Anna A. Brezgunova, Irina B. Pevzner, Ljubava D. Zorova, Vasily N. Manskikh, Vasily A. Popkov, Denis N. Silachev, Dmitry B. Zorov

**Affiliations:** 1 A.N. Belozersky Institute of Physico-Chemical Biology, Lomonosov Moscow State University, 119992 Moscow, Russia; irinapevzner@mail.ru (I.B.P.); lju_2003@list.ru (L.D.Z.); manskikh@mail.ru (V.N.M.); popkov.vas@gmail.com (V.A.P.); silachevdn@belozersky.msu.ru (D.N.S.); 2 V.I. Kulakov National Medical Research Center of Obstetrics, Gynecology and Perinatology, 117997 Moscow, Russia; 3 Institute of Molecular Medicine, Sechenov First Moscow State Medical University, 119991 Moscow, Russia; 4 Biological Faculty, Lomonosov Moscow State University, 119992 Moscow, Russia; elqu7@yandex.ru; 5 Faculty of Bioengineering and Bioinformatics, Lomonosov Moscow State University, 119992 Moscow, Russia

**Keywords:** kidney injury, renal failure, endotoxin, sepsis, age, oxidative stress, fibrosis, regeneration

## Abstract

Neonatal sepsis is one of the major causes of mortality and morbidity in newborns, greatly associated with severe acute kidney injury (AKI) and failure. Handling of newborns with kidney damage can be significantly different compared to adults, and it is necessary to consider the individuality of an organism’s response to systemic inflammation. In this study, we used lipopolysaccharide (LPS)-mediated acute kidney injury model to study mechanisms of kidney cells damage in neonatal and adult rats. We found LPS-associated oxidative stress was more severe in adults compared to neonates, as judged by levels of carbonylated proteins and products of lipids peroxidation. In both models, LPS-mediated septic simulation caused apoptosis of kidney cells, albeit to a different degree. Elevated levels of proliferating cell nuclear antigen (PCNA) in the kidney dropped after LPS administration in neonates but increased in adults. Renal fibrosis, as estimated by smooth muscle actin levels, was significantly higher in adult kidneys, whereas these changes were less profound in LPS-treated neonatal kidneys. We concluded that in LPS-mediated AKI model, renal cells of neonatal rats were more tolerant to oxidative stress and suffered less from long-term pathological consequences, such as fibrosis. In addition, we assume that by some features LPS administration simulates the conditions of accelerated aging.

## 1. Introduction

Neonatal sepsis, when it occurs at up to 28 days of age, is one of the major causes of mortality and morbidity in newborns. According to the World Health Organization, in 2010, more than 1.3 million newborns in the United States died before reaching 28 days of age because of infections [1,2]. Sepsis is commonly considered a complex clinical syndrome, which is mostly defined by severe systemic inflammation leading to multiple pathologies and organs dysfunction and failure [3,4]. The immune system is considered to be a key player in sepsis development due to its association with infection (bacterial or fungal). However, the immune system of newborns is known to mature only at 24 weeks [5], being underdeveloped before this term. This fact heavily affects sepsis onset and propagation in neonates, thus making diagnostics and treatment of sepsis and SIRS (systemic inflammatory response syndrome) in neonates quite different from adults [6].

The immature immune system of infants is poorly studied and understood, and it is extremely difficult for clinicians to predict responses to both inflammation-inducing agents and treatment [7]. For example, term newborns usually react to sepsis by elevated temperature (more than 37.8 °C), but preterm newborns more often react by hypothermia due to their underdeveloped temperature control system [8]. It is also known that neutrophils activity [9] and concentrations of IgG [9,10] are low in neonates. Monocytes and macrophages of neonates contain less toll-like receptor 4 (TLR4) [11,12,13,14], and their response to antigens and endotoxin is retarded, thus yielding low production of proinflammatory cytokines. The complement system in neonates is under-regulated as well [15]. In concert, these factors lead to newborns being more susceptible to infections, which increases both the chance of sepsis development and its severity [16]. On the other hand, these features can be beneficial against the inflammatory aspect of sepsis pathogenesis, which is the leading damaging factor.

Lastly, the mother’s health should also be considered. It is common for neonates to receive infection from their mothers, and the mother’s inflammatory response could strongly affect the fetus, with further complications occurring after birth [17].

The kidney is one of the main organs that suffer from sepsis. Sepsis triggers 50% of all acute kidney injury (AKI) cases in critically ill patients, and its mortality rate reaches 60% [18,19,20]. Moreover, in neonates, due to their immature immune system, the situation is usually complicated by administered antibiotics, which are required to fight infection but more likely to causing damage to the kidneys. Antibiotic therapy can complicate the dysfunction of many organs, but the kidney becomes the most vulnerable organ because of the well-known nephrotoxicity of antibiotics [21,22,23]. Since kidney damage in systemic inflammatory response can arise both from infectious and noninfectious causes and can include hyperactive or hypoactive inflammation phases, this leads to a new view on sepsis management that is based on consideration of key points of the process, including mitochondria dysfunction and changes in redox homeostasis. This is not only a new approach for therapy but the only one which is considered to be ubiquitous to all sepsis patients as it addresses the mechanism of cellular damage directly. Recently, the existence of the “sepsis redox cycle” was postulated by researchers [24]. According to this cycle, interleukins (IL6, IL8) activate NF-κB (nuclear factor κB), which upregulates inducible nitric oxide (NO) synthase (iNOS). This leads to elevated NO levels, which inhibit cytochrome oxidase and thus decelerate mitochondrial electron transport, resulting in increased production of superoxide anion radical and hydrogen peroxide in mitochondria [25]. Hydrogen peroxide further activates NF-κB, which closes this positive self-amplifying loop, ultimately yielding mitochondria, cell, and organ dysfunction. This is highly applicable to the kidney, where damage during sepsis is associated with mitochondria dysfunction, and where reactive oxygen species (ROS) generation [26]—with ROS generation as a self-promoting loop [27]—might be terminated on initial stages by right approaches.

The possibility of treating ROS-associated kidney pathologies through addressed maintenance of redox homeostasis reached by different agents, like antioxidants, looks very promising. We have also demonstrated the efficiency of this approach on adult kidney damage models in our earlier work [28,29]. However, for neonatal kidneys, the method requires careful study of oxidative stress development under septic conditions with a proper comparison to those in adult kidneys. In this study, we have chosen lipopolysaccharide (LPS)-mediated acute kidney injury as a model for our studies because it triggers the same mechanisms as natural sepsis does and ultimately results in the same mitochondria-mediated damage. At the same time, it is much more controllable and reproducible than bacterial models. This study aims to compare AKI, particularly the associated oxidative damage in neonatal and adult rats during LPS-mediated sepsis simulation, and to identify mechanisms of kidney cells damage following AKI that is common to both groups.

## 2. Materials and Methods

### 2.1. Animal Experiments

**Figure 1:**
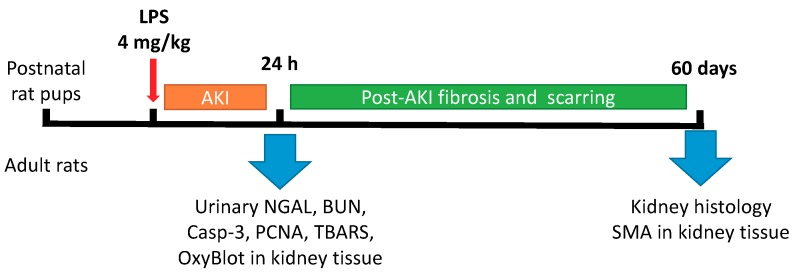
Design of experiments with lipopolysaccharide (LPS)-mediated acute kidney injury (AKI) in adult and newborn rats. NGAL: neutrophil gelatinase-associated lipocalin 2; BUN: blood urea nitrogen; PCNA: proliferating cell nuclear antigen; TBARS: thiobarbituric acid-reactive substances; SMA: α-smooth muscle actin.

The animal protocols used in this work were evaluated and approved by the institutional animal ethics committee (Protocol 9/17 from 28 April 2017) in accordance with Federation for Laboratory Animal Science Associations (FELASA) guidelines. The experiments were performed on outbred white rats. Dams and their pups were kept in cages with a temperature-controlled environment (23 ± 2 °C) with light on from 9 a.m. to 9 p.m. Dams had ad libitum access to food and water, and pups were checked daily for health. The number of pups in a litter was 9–12. Endotoxin-mediated AKI was performed on 3-days-old neonatal and adult rats by intraperitoneal administration of LPS from *E. coli* (0127:B8 strain, Sigma Aldrich, St. Louis, MO, USA) in concentration 4 mg/kg; the control group was administrated with saline infusion. The total number of animals in each group was 6 (from different litters). The experimental design is presented in Figure 1. Briefly, 24 h after LPS administration, blood, urine and kidneys were taken from animals. Urine samples were diluted 1:1 with 2× sample buffer and boiled for 5 min and then centrifuged at 12,000× *g* for 3 min. Blood samples were taken to determine blood urea nitrogen (BUN) using AU480 Chemistry System (Beckman Coulter, Brea, CA, USA).

### 2.2. Western Blotting

Kidneys were homogenized in 1 mL of phosphate buffered saline (PBS) with 1 mM of protease inhibitor phenylmethylsulfonyl fluoride (PMSF), then centrifuged at 1000× *g* for 3 min. Five microliters of the supernatant were used to measure protein concentration using the bicinchoninic acid kit (Sigma Aldrich, St. Louis, MO, USA); the rest was taken for blotting analysis to evaluate the proliferative potential, protein oxidation, and apoptotic activity. Samples were loaded onto 15% Tris-glycine polyacrylamide gels (10 μg protein per lane). After electrophoresis, gels were blotted onto polyvinylidene difluoride (PVDF) membranes (Amersham Pharmacia Biotech, Amersham, UK). Membranes were blocked with 5% non-fat milk in PBS/0.1% Tween-20 and subsequently incubated with primary rabbit antibodies to neutrophil gelatinase-associated lipocalin 2 (NGAL) 1:1000 (Abcam, Cambridge, UK), proliferating cell nuclear antigen (PCNA) 1:1000 (13110, Cell Signaling Technology, Danvers, MA, USA), and to caspase-3 1:500 (9661, Cell Signaling Technology, Danvers, MA, USA). Fibrosis formation was examined two months after a single LPS administration using rabbit antibodies for α-smooth muscle actin (SMA) 1:500 (14968, Cell Signaling Technology, Danvers, MA, USA). Carbonylated proteins (protein oxidation marker) were measured using OxyBlot kit (S7150 OxyBlot Protein Oxidation DetectionKit, Millipore, Burlington, MA, USA). Membranes were processed with secondary antibodies conjugated with horseradish peroxidase 1:10,000 (IMTEK, Moscow, Russia). Detection was performed by ChemiDoc™ MP imaging system (BioRad, Hercules, CA, USA) with WesternBright™ Enhanced Chemiluminescence kit (Advansta, Menlo Park, CA, USA).

### 2.3. Renal Histology

After sacrificing the animal, the kidneys were immediately taken, fixed in a 10% neutral buffered formalin solution, embedded in paraffin, and used for histopathological examination. Five-micrometer-thick sections were cut, deparaffinized, hydrated, and stained with Masson trichrome [30]. The renal sections were examined for fibrosis in an automated fashion by quantification of a specific color in the kidney tissue using ImageJ (National Institutes of Health, Bethesda, MD, USA) color threshold plugin. Color threshold settings were adjusted manually for each image. Percentage of the field of view with positive staining was considered to be fibrosis ratio. A minimum of seven fields of view for each kidney slice was examined.

### 2.4. Measurement Thiobarbituric Acid-Reactive Substances (TBARS)

TBARS in kidney tissue homogenates were explored by a conventional colorimetric method with the use of malondialdehyde–thiobarbituric acid reaction according to Mihara and Uchiyama [31]. Briefly, each sample was mixed with 0.8% thiobarbituric acid and 1% H_3_PO_4_ in the ratio by volume 0.9:1.0:3.0. The mixture was boiled for 45 min, cooled to room temperature, and then centrifuged at 15,000× *g* for 10 min. The absorbance of the resulting supernatant at 532 nm was measured using a Hitachi 557 spectrophotometer. 1,3,3-tetraetoxipropan (Sigma Aldrich, St. Louis, MO, USA) was used as a calibration standard. The content of thiobarbituric acid (TBA)-reactive products was finally normalized by total protein content.

### 2.5. Statistics

Statistical analyses were performed using STATISTICA 7.0 for Windows (StatSoft, Inc., Palo Alto, CA, USA). Values are given as mean ± standard error of the mean (SEM). Variance homogeneity was assessed with Levene’s test. Statistical differences were analyzed using the Kruskal–Wallis test with the Mann–Whitney *u*-test (the Bonferroni post hoc correction was applied). Differences were considered significant at *p* ≤ 0.05.

## 3. Results

### 3.1. Oxidative Stress in Kidneys of Neonatal and Adult Rats

**Figure 2:**
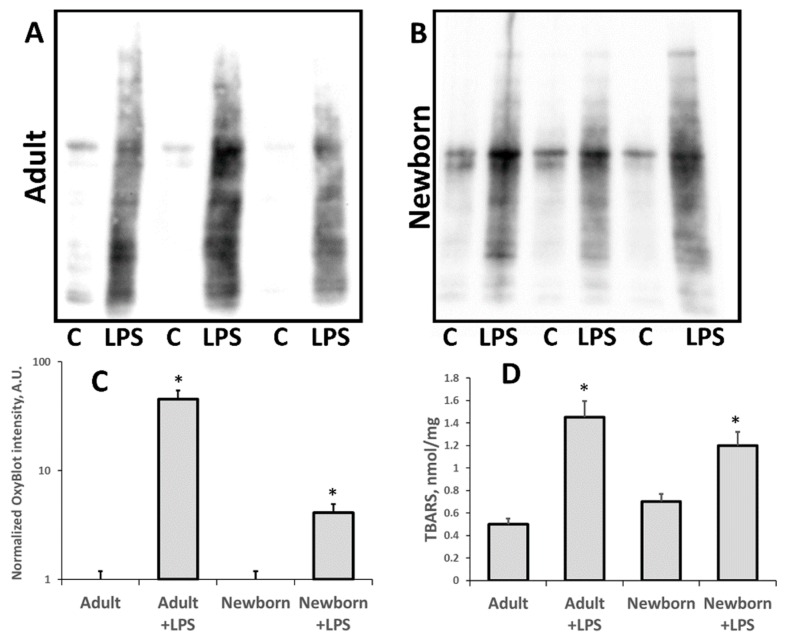
Oxidative stress in neonatal and adult kidneys 24 h after LPS treatment. Estimation of carbonylated proteins in kidney tissue of (**A**) adult rats and (**B**) newborn rats. (**C**) Densitometry of OxyBlot^®^ plots. (**D**) Analysis of lipid peroxidation in kidneys as measured by thiobarbituric acid-reactive substances (TBARS) accumulation. Number of animals for Oxyblot densitometry and TBARS analysis: Adult (*n* = 6), Newborn (*n* = 6), Adult + LPS (*n* = 5), Newborn + LPS (*n* = 5). *: *p* < 0.05.

We analyzed the development of oxidative stress and accumulation of oxidative modified (carbonylated) proteins and lipids (TBARS) in 3-days-old neonates and adult rats (Figure 2). After 24 h of LPS administration, we observed much higher levels of carbonylated proteins in both adult and neonatal rats compared to the relevant control. However, in neonatal AKI kidneys this increase was smaller compared to the adult AKI group. While in adult kidneys, the signal of OxyBlot was about 20 times higher after LPS treatment, the same signal rose only about two times in neonatal kidneys (Figure 2C). Note that while adult rats demonstrated a higher increase in carbonylated proteins level, the intact neonatal kidneys initially had a slightly higher Oxyblot signal than the intact adult kidneys. Our results indicate that oxidative stress during LPS-induced, sepsis-like kidney injury is more severe in adults than in neonates.

TBA-reactive products reporting on the content of the main product of lipid oxidation—malondialdehyde (MDA)—as well as some other lipid peroxides are other markers of oxidative damage in kidney cells. We found (Figure 2D) that similar to carbonylated proteins, TBARS levels in kidney tissue were much higher after LPS injection, both in adult and neonatal rats compared to intact controls. However, in neonatal AKI kidneys, this increase was less than in adult AKI group. Thus, our results indicate that LPS-mediated sepsis indeed leads to oxidative stress and that oxidative kidney tissue damage is less severe in neonates.

### 3.2. Changes Associated with LPS-Mediated Oxidative Stress

**Figure 3:**
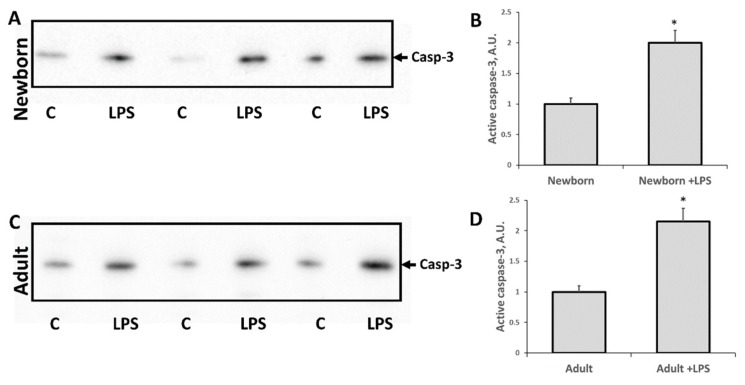
Induction of apoptosis in neonatal and adult kidneys 24 h after LPS treatment. Immunoblots for cleaved procaspase-3 (caspase-3, i.e., mature, active form) levels in kidneys of (**A**,**B**) neonatal rats and (**C**,**D**) adult rats. Densitometry analysis of bands in newborn and adults are presented in (**B**) and (**D**), respectively. Number of animals for western blot densitometry: Adult (*n* = 6), Newborn (*n* = 6), Adults + LPS (*n* = 5), Newborn + LPS (*n* = 5). *: *p* < 0.05.

It is well known that oxidative stress can induce cell death of different nature, including apoptosis. To evaluate apoptosis activation in kidney cells in response to LPS administration, we measured levels of active caspase-3 using western blotting. Twenty-four hours after LPS injection, levels of cleaved (active) caspase-3 in both adult and neonatal rats were increased two times vs. relevant intact groups (Figure 3). There was no significant difference in apoptosis activation in response to LPS-mediated injury between adults and neonates.

**Figure 4:**
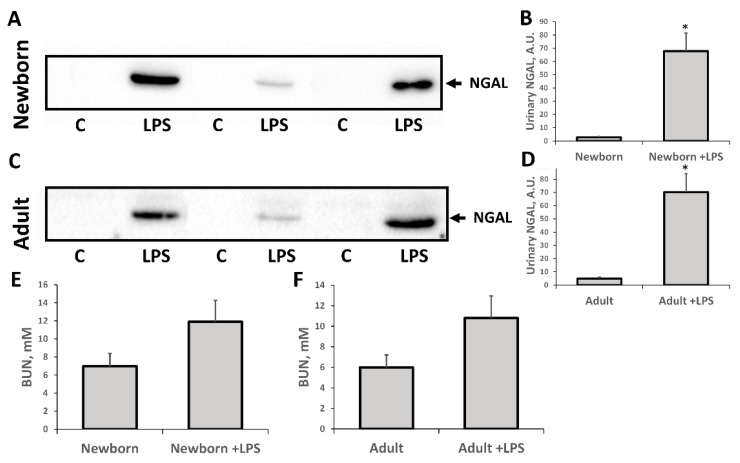
Acute kidney injury in (**A**,**B**,**E**) neonatal rats and (**C**,**D**,**F**) adult rats 24 h after LPS treatment. Significant increase in urinary neutrophil gelatinase-associated lipocalin 2 (NGAL) content and some rise of blood urea nitrogen (BUN) was observed. Diagrams on B, D represent densitometry analysis of corresponding bands on the blots. Number of animals for western blot densitometry: Adult (*n* = 4), Newborn (*n* = 4), Adult + LPS (*n* = 4), Newborn + LPS (*n* = 4). *: *p* < 0.05.

We demonstrated that LPS injection led to acute kidney damage. Twenty-four hours after administration of LPS, a significant increase in NGAL (highly sensitive biomarker of AKI) was observed in the urine of neonatal and adult rats (Figure 4). In addition, there was a trend of increasing BUN in both experimental groups (Figure 4E,F) indicating some renal failure. While only slight renal failure was observed by this indicator after exposure to LPS, the AKI marker NGAL in urine demonstrated the presence of severe AKI in neonatal and adult rats in the first 24 h.

**Figure 5:**
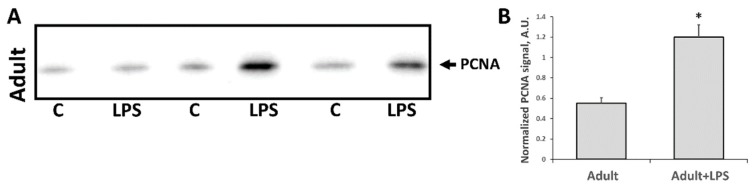

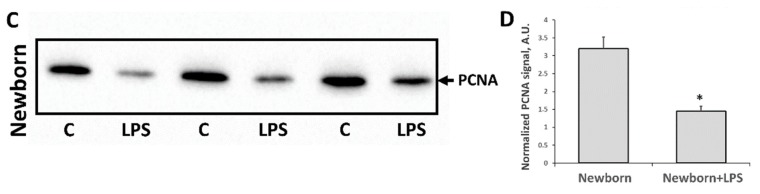
Proliferative activity of kidney cells of newborn and adult rats 24 h after LPS treatment. Immunoblots for proliferating cell nuclear antigen (PCNA) levels in (**A**) adult kidneys and (**C**) neonatal kidneys and their densitometry analysis in (**B**,**D**), respectively. Number of animals for western blot densitometry: Adult (*n* = 6), Newborn (*n* = 6), Adult + LPS (*n* = 5), Newborn + LPS (*n* = 5). *: *p* < 0.05.

Another marker associated with kidney injury and regeneration after damage is the level of proliferation marker PCNA. Since cell proliferation is almost absent in adult kidneys, it is believed that injury activates PCNA expression as part of a signal for regeneration. Indeed, in our study, PCNA levels in adult kidneys were significantly increased 24 h after LPS administration (Figure 5A,B). However, in neonates, the response was opposite (Figure 5C,D), possibly due to the fact that until the seventh day after birth, the kidney is still developing with proper proliferative activity. PCNA level in intact neonates kidneys was already much higher compared to the adult ones (compare Figure 4A,C) as described earlier [32]. LPS-mediated injury caused a drop of PCNA levels in neonatal rats, which was the opposite of the adult kidneys’ response.

### 3.3. Fibrosis Formation after Acute Kidney Injury

**Figure 6:**
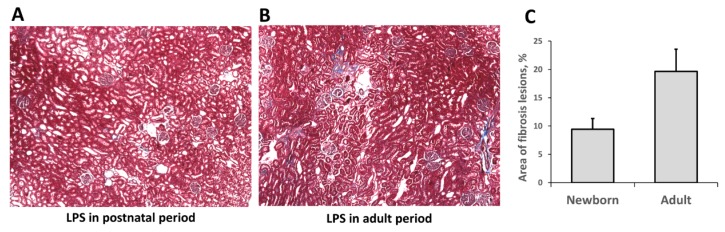
Histological evaluation of fibrotic changes in neonatal and adult kidneys two months after LPS treatment. (**A**) Kidneys of rats that received LPS in neonatal age and (**B**) kidney of rats treated with LPS in adult age. (**C**) Evaluation of fibrotic lesions revealed increased fibrosis in adult group. Number of animals: Adult (*n* = 3), Newborn (*n* = 3).

**Figure 7:**
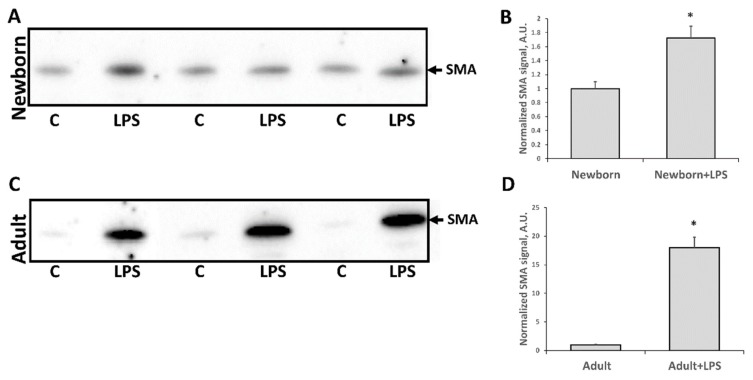
Kidney fibrosis, estimated by α-smooth muscle actin (SMA) levels in kidney tissue two months after LPS treatment. (**A**,**B**) Immunoblots of kidneys of rats that received LPS in neonatal age and (**C**,**D**) rats treated with LPS in adult age. (**B**,**D**) Densitometry analysis of bands in neonates and adults, respectively. Number of animals for Western blot densitometry: Adult (*n* = 6), Newborn (*n* = 6), Adults + LPS (*n* = 5), Newborn + LPS (*n* = 5). *: *p* < 0.05.

One of the most severe consequences of any AKI is a fibrosis formation, i.e., replacement of functional kidney tissue by fibroblasts and connective tissue scarring. We evaluated fibrosis formation two months after LPS administration using classical histological approach (Figure 6). Connective tissue (collagen formations) was found in both LPS-treated groups (those that received LPS in infancy or adult age) after two months, indicating that fibrosis formation had taken place. We found more pronounced renal fibrotic scaring in the adult group compared to neonatal kidneys, as shown in the digital image analysis (Figure 6C). Moreover, α-smooth muscle actin (SMA) levels—one of the well-established markers for fibrosis formation in kidneys—demonstrated significant differences (Figure 7). SMA levels in adult kidneys two months after LPS injection were significantly higher compared to intact animals. In kidneys of rats treated with LPS in neonatal age, SMA levels were higher as well but not as much as in adult kidneys. Overall, these results indicate that fibrosis formation took place two months after LPS-induced AKI and that SMA accumulation was less in neonatal kidneys than in the adult one.

## 4. Discussion

Most biological studies of sepsis and AKI were carried out on adult animals. However, translating experimental results from bench to bedside is difficult because for a wide spectrum of diseases, adults are the least presented patients in clinics and hospital beds, while children and old people are the most often presented group. Sepsis is one such pathology that affects neonates more often [2] and has pathogenesis that is quite different in neonates compared to adults or elders. Sepsis is a complex condition, which includes heterogeneous interconnected processes—bacterial invasion, inflammation, and oxidative stress—that are all highly affected by neonatal status. An immature immune system compromises an organism’s ability to fight a disease in case of bacterial sepsis. On the other hand, it is not clear how underdeveloped immune system affects inflammatory component of sepsis, i.e., whether it relieves or burdens the pathology. Finally, neonates are believed to have deficient antioxidant systems [33,34,35,36], possibly due to the fact that intrauterine fetuses are exposed to three times lower O_2_ concentration than after birth (pO_2_ 30 and 90 mm Hg, respectively) [37]. Levels and activity of antioxidant enzymes superoxide dismutase and glutathione peroxidase and other antioxidants are low in neonate plasma, making neonates potentially more vulnerable to pathologies associated with oxidative stress [33,38,39]. Therefore, the antioxidant system in the first few days after birth does not correspond to the environmental oxygen challenge. However, neonates also have lower capacities to produce ROS [40], and this feature could affect oxidative stress-associated pathologies outcomes as well. Weaker antioxidant system could complicate the course of septic damage in newborns, whereas weaker ROS production may have a beneficial effect.

In our study, we found the last assumption to be true for AKI—both carbonylated proteins levels and TBARS after LPS-mediated kidney injury were higher in adults than in neonates. This indicates that oxidative stress in septic kidneys is lower in neonates. A possible explanation of decreased oxidative damage is the underdeveloped immune response of newborns, i.e., underexpression of TLR4 interacting with LPS. Thus, the immature immune system in neonates might afford some protection by preventing the initiating steps of “sepsis redox cycle”, which triggers oxidative damage in adults.

Considering the mechanisms of kidney injury in sepsis, we should mention three main approaches for modeling sepsis: LPS injection, bacteria injection, and caecal ligation and puncture (CLP) [41]. All of them have their specificity and disadvantages and address different aspects of sepsis pathogenesis. LPS injection is the most reproducible and simple model as it allows exploring of basic processes such as inflammation and oxidative damage by bypassing bacteria-related factors, such as toxins production. Moreover, the LPS model is more relevant for sepsis-like conditions like SIRS. Bacteria injection and CLP are more appropriate models for clinical sepsis pathogenesis but are less reproducible and more heterogeneous, making it harder to study molecular mechanisms in these models. Also, bacterial toxins and antibiotics administration could dramatically affect the flow and outcome of the pathology. Since the aim of our work was to address oxidative damage and antioxidant capacities in neonates, we studied LPS-mediated AKI and revealed that oxidative damage per se was less severe in neonates.

Regarding the mechanisms of development of AKI in LPS-simulated sepsis, it is worth noting the increase in the cleaved procaspase-3, which is a marker of apoptosis induction. The observed increase in the level of this protein in the kidney indicates that the endotoxin action causes apoptotic death of the kidney cells. Note that at the same time, the AKI marker NGAL in the urine is increased. Thus, it can be assumed that kidney damage is associated with apoptosis of the tubular cells. However, between the groups of newborns and adult rats, there was no difference in the manifestation of these two parameters when exposed to LPS.

We also observed an increase in PCNA levels in adult kidney tissues and a reduction in neonatal kidneys after AKI. PCNA elevation is commonly associated with activation of tubular regeneration, and it has been well described that AKI activates regeneration in the adult kidney [42]. However, basal levels of PCNA in kidneys of intact newborn rats is essentially higher compared to adults. We believe that even after an LPS-induced drop of PCNA in neonates, the proliferation remains will be higher or equal to AKI-induced increased level of PCNA in adults. We speculate that this is due to the ongoing development of kidney in neonates, which is associated with tubular cells proliferation that keeps the basal level of PCNA elevated. Thus, LPS exposure affects these proliferating cells and causes a drop in their number because of cell death. However, we could give an alternative explanation of different reaction of PCNA level in adult and newborn kidney. PCNA is a marker of proliferation, and fibroblasts proliferation during fibrosis development could also lead to an increase of this protein. In our study, we demonstrated fibrotic scaring after LPS-mediated kidney injury as indicated by collagen appearing in kidney tissue two months after AKI. Moreover, SMA levels were much higher in adults after AKI compared to neonates, which reflects progressing fibroblasts proliferation and transformation to myofibroblasts [43]. Thus, the possibility exists that PCNA increase in adult kidney tissue can be associated to some extent with fibroblast proliferation and that it is a part of fibrosis formation rather than just being a sign of regeneration.

## 5. Conclusions

Our data suggest that in the model of LPS-mediated AKI, neonatal rats kidney are more tolerant to oxidative stress, with less oxidative damage to renal cell and less long-term pathological consequences like fibrosis. We propose that it could be partially due to the immature immune system of newborns, which is the main player contributing to oxidative stress associated with sepsis in adults. We emphasize that the key component of sepsis development—oxidative stress—is still poorly described in neonates, at least in relation to the kidney. We need a comprehensive understanding of sepsis impact on susceptible organs of neonates, such as the kidney, to create specific approaches for treating this dangerous pathology in newborns and infants.
